# Präoperative Radiotherapie plus Resektion vs. Operation allein bei Patienten mit primärem retroperitonealem Sarkom (EORTC-62092: STRASS): eine multizentrische, randomisierte Phase-III-Studie

**DOI:** 10.1007/s00066-020-01734-5

**Published:** 2021-01-05

**Authors:** Gerhard G. Grabenbauer

**Affiliations:** 1 Strahlentherapie & Radioonkologie am Klinikum Coburg, Ketschendorfer Straße 33, 96450 Coburg, Deutschland; 2 Universitätsklinikum Erlangen, Erlangen, Deutschland

## Hintergrund und Ziele

Im Gegensatz zur Situation beim Extremitätensarkom gilt der Stellenwert der Radiotherapie bei einem retroperitonealen Sarkom als weniger gesichert. Ziel dieser Studie war es daher, den Nutzen einer präoperativen Bestrahlung (RT), gefolgt von einer Resektion (Op.), im Vergleich zur alleinigen Op. zu untersuchen [1].

## Patienten und Methoden

Die EORTC-62092-Studie [1] randomisierte erwachsene Patienten mit einem histologisch gesicherten retroperitonealen Sarkom in zwei Behandlungsarme: Sofortige Op. vs. präoperative RT mit 50,4 Gy in 28 Fraktionen (meist IMRT), gefolgt von einer Op. nach 4–8 Wochen. Der primäre Endpunkt war die Überlebensrate ohne abdominelles Rezidiv.

## Ergebnisse

Von 2012 bis 2017 wurden von insgesamt 31 Institutionen 266 Patienten rekrutiert und randomisiert, 133 in jeden Arm. Die mediane Nachbeobachtungszeit betrug 43 Monate. Die Verteilung der histologischen Subtypen war in beiden Armen ähnlich: Liposarkome 74–75 %, Leiomyosarkome 12–17 %, andere 8–14 %. Zu ca. 30 % lagen Low-grade-Sarkome vor, zu ca. 45 % Intermediate- und High-grade-Sarkome. Tatsächlich hatten 96 % der Patienten im alleinigen Op.-Arm die radikale Resektion und 89 % der Patienten im Kombinationsarm die RT mit nachfolgender Op. Perioperative Komplikationen wurden bei 27 % nach alleiniger Op. und bei 37 % der Patienten im Kombinationsarm gesehen (Transfusionsrate: 19 % vs. 29 %).

In der Intention-to-treat-Analyse (ITT) betrug die mediane rezidivfreie Überlebenszeit nach alleiniger Op. 5 Jahre und im RT + Op.-Arm 4,5 Jahre (HR 1,01, 95 %-CI 0,71–1,44, *p* = 0,95). Entsprechend waren auch die „3-Jahres-Überlebensraten ohne abdominelles Rezidiv“ gleich (58,7 % vs. 60,4 %).

Nach Ausschluss von 19 Patienten (sog. „Post-hoc-Analyse“), die unter der Radiotherapie progredient waren (aber dennoch komplett operiert wurden) oder „unfit“ wurden, waren die 3‑Jahres-Überlebensraten mit 62,2 % vs. 71,3 % höher im Kombinationsarm (HR 0,78, 95 %-CI 0,53–1,16). In der Subgruppe der Liposarkome waren insgesamt 65 Rezidive zu verzeichnen (39 nach alleiniger Op. vs. 26 nach RT + Op.), was sich in einer verbesserten 3‑Jahres-Überlebensrate mit 65 % auf 76 % niederschlug (HR 0,62, 95 %-CI 0,38–1,02).

## Schlussfolgerung der Autoren

Die präoperative RT sei bei retroperitonealen Sarkomen keine Standardbehandlung.

## Kommentar

Retroperitoneale Sarkome sind eine seltene und zusätzlich noch relativ heterogene Tumorentität. Umso verdienstvoller ist es, qualitativ hochwertige Daten zur Evidenz verschiedener Behandlungsmodalitäten zu generieren, was der EORTC mit der hier vorgelegten STRASS-Studie gelungen ist. Zweifelsohne ist – rein formal gesehen – die Schlussfolgerung der Autoren, der zufolge der präoperativen Radiotherapie kein Stellenwert zuzumessen sei, als korrekt anzusehen. Aus radioonkologischer Sicht allerdings bedarf es einer kritischen Stellungnahme.

1. *Nachbeobachtungszeit zu knapp*
  Mit lediglich 3,6 Jahren ist die mediane Nachbeobachtungszeit zur wirklichen Beurteilung des Langzeitverlaufs eines retroperitonealen Sarkoms und damit zur Definition des Stellenwerts einer Radiotherapie zu knapp. Größere (wenn auch retrospektive) Studien [2] zeigen, dass zwar die Rate an Fernmetastasen mit 21 % (95 %-CI 19,0–24,6) nach 5, 8 und 10 Jahren stabil blieb, jedoch die Lokalrezidivraten stetig anstiegen: Nach 5 Jahren betrug die kumulative Inzidenz 25,9 % (95 %-CI 23,1–29,1), nach 8 Jahren 31,3 % (95 %-CI 27,8–35,1) und nach 10 Jahren 35 % (95 %-CI 30,5–40,1). Dieser Sachverhalt deckt sich mit den klinischen Erfahrungen.
2. *Fehlende Stratifikation nach Histologie und Grading*
  Eine Subgruppenanalyse wird seitens der Autoren nicht angeboten, da offenbar keine Stratifikation nach histologischer Entität und Grading vorgesehen war. Es wurde zwar nach WHO-Performance-Status und Institution, jedoch nicht nach den genannten Kriterien stratifiziert. Dennoch bleibt das positive Signal in der Subgruppe der Patienten mit Liposarkom, welches eine Halbierung der Lokalrezidive nach der Kombinationsbehandlung aus RT und Op. im Vergleich zur alleinigen Op. zeigt.
3. *Zielvolumendefinition und Rezidivmuster*
  Das Studienprotokoll sah eine Festlegung des klinischen Zielvolumens (CTV) als „geografische Expansion“ des makroskopischen Tumorvolumens (GTV) um 5–6 mm vor, was fraglos für ein aggressives Sarkom als zu knapp angesehen werden muss. Zudem werden in der Publikation keine Daten zum etwaigen Rezidivmuster präsentiert, was in diesem Licht besonders von Interesse gewesen wäre und den geringen Effekt der RT erklären könnte.
4. *Erhöhte Toxizität*
  Die Autoren beschreiben relativ hohe Raten an akuter Toxizität vom CTC-Grad 3–4, so z. B. eine Lymphopenie bei 77 %, eine Anämie bei 12 % und eine Hypalbuminämie bei 12 % nach RT und Op. Diese waren nach alleiniger Op. deutlich geringer ausgeprägt. Eine wirkliche Per-protocol-Behandlung fand demnach bei 128/133 (96 %) im Op.-Arm, aber nur bei 119/133 (89 %) der Patienten im RT + Op.-Arm statt, was ggf. auch die ungünstigen Ergebnisse der ITT-Analyse erklärt. In jedem Fall sollte eine hinreichende supportive Therapie im Zusammenhang mit der abdominellen Radiotherapie der relativ großen retroperitonealen Volumina geboten sein, zumal andere Autoren [3] von einer a priori bestehenden Malnutrition bei mindestens 50 % der Patienten berichten.

## Fazit

Rein formal kann durchaus die präoperative RT in der Subgruppe der retroperitonealen Liposarkome auf der Basis der hier diskutierten Daten empfohlen werden. Im Übrigen bleibt die gesicherte Indikation [4, 5] zur postoperativen RT (ggf. unter Einschluss der intraoperativen RT) davon unberührt.

*Gerhard G. Grabenbauer, Coburg*
